# A New Modified Twist Drill Craniostomy Using a Novel Device to Evacuate Chronic Subdural Hematoma

**DOI:** 10.1097/MD.0000000000003036

**Published:** 2016-03-11

**Authors:** Qing-Feng Wang, Cheng Cheng, Chao You

**Affiliations:** From the Department of Neurosurgery, West China Hospital, West China School of Medicine, Sichuan University, Chengdu (Q-FW); Department of Neurosurgery, The Second Clinical School, Yangzhou University, Yangzhou (CC); and Department of Neurosurgery, West China Hospital, Sichuan University, Chengdu (CY), China.

## Abstract

Compared with burr hole craniostomy (BHC), twist drill craniostomy (TDC) is increasingly popular because of its minimal invasiveness in evacuating chronic subdural hematoma (CSDH). However, the TDC technique varies and is continually developing; moreover, no consensus yet exists regarding the optimal protocol, and the efficacy and safety of TDC is still controversial, especially with respect to a specific method. This article introduces a new modified TDC technique using a novel device, the YL-1 puncture needle, and evaluates its efficacy and advantages compared with BHC.

A retrospective study involving 121 patients with CSDH who underwent surgery at a single center was conducted, involving 68 patients undergoing modified TDC (TDC group) and 53 patients treated by BHC (BHC group). The neurological outcome was studied to evaluate the surgery efficacy, and the radiological outcome was assessed as a supplement to the surgery efficacy. In addition, complications, recurrence, and reoperation, as well as pneumocrania, operation duration, and length of stay, were studied to evaluate the advantages of the modified TDC compared with BHC. Independent sample *t* tests or rank-sum tests were used to compare the outcomes between the 2 groups.

The neurological and radiological outcomes did not differ significantly between the TDC and BHC groups (*P* = 0.852 and *P* = 0.232, respectively), while the rates of complication and pneumocrania in patients who underwent the modified TDC were significantly lower than that in those who underwent BHC (*P* = 0.021 and *P* < 0.001, respectively). The recurrence and reoperation rates in patients from the 2 groups were similar (*P* = 0.566 and *P* = 0.715, respectively). The operation duration and length of hospital stay of the patients who underwent the modified TDC were significantly shorter than those of the patients who underwent BHC (both *P* < 0.001).

Modified TDC with a YL-1 puncture needle is a minimally invasive surgical technique to treat CSDH; this procedure is as effective as BHC, but safer and simpler than BHC, and should be considered for patients with CSDH, especially the elderly.

## INTRODUCTION

Chronic subdural hematoma (CSDH) is a common neurological condition that mainly occurs in the elderly. The minimally invasive procedure—twist drill craniostomy (TDC)—is theoretically more suitable to treat CSDH than the traditional procedure—burr hole craniostomy (BHC). However, whether TDC or BHC is the optimal strategy is still controversial. First, the TDC technique varies, and there is no consensus on the optimal technique. Second, it is unclear whether the surgical efficacy of TDC is reliable. Although some studies have demonstrated that TDC is as effective as BHC,^1–4^ which was considered to be the most efficient choice for surgical drainage during uncomplicated CSDH,^5^ the existing evidence is still unsubstantial to recommend TDC as a first-line strategy, especially as it comes to a specific technique. The third question is whether TDC is actually minimally invasive and safe? In fact, TDC is not free from complications, such as inadequate drainage, brain penetration, acute epidural hematoma, and catheter folding.^6^

Here, we present a modified TDC technique that employs a novel device (the YL-1 puncture needle) and evaluates its efficacy and advantages through a retrospective comparison with classic BHC.

## METHODS

### Patients

We retrospectively reviewed 121 patients undergoing surgery for CSDH from September 2009 to November 2014 at the Department of Neurosurgery of the Second Clinical School of Yangzhou University, Jiangsu, China. From September 2009 to July 2012, 53 patients with CSDH underwent BHC. After the novel TDC technology was introduced to this department, 68 patients underwent modified TDC. Thus, the patient population was divided into 2 groups: a BHC (n = 53) group and a TDC (n = 68) group. Neurological status was evaluated as the primary outcome. The secondary outcomes included radiological features, complication, recurrence, reoperation, pneumocrania, operation duration, and length of hospital stay. Both the Ethics Committees of the West China Hospital of Sichuan University and the Second Clinical School of Yangzhou University deemed that an ethical review was not needed for this retrospective analysis.

Symptomatic patients with CSDH (diagnosed based on a computed tomography [CT] scan to have a typical crescent shape, as well as an isodense, hypodense, or heterogeneous hematoma that exerted a mass effect on midline structures) underwent surgery. The patients were preoperatively evaluated at admission and postoperatively evaluated at discharge. These evaluations assessed neurological symptoms (headache, weakness, cognitive decline, and consciousness) and radiological features (hematoma size and extent of midline shift). The largest width of the hematoma was considered to be the size of the hematoma and was obtained via CT or magnetic resonance imaging (MRI). Patients showing the reappearance of a hyperdense crescent-shaped hematoma or increasing hematoma cavity volume on the operated side on a CT scan within a few months after surgery were considered to have experienced recurrence; symptomatic hematomas with a significant mass effect underwent repeated surgeries.

### Surgical Procedures

Senior neurosurgery residents or more experienced surgeons performed the procedures. BHC was primarily performed under general anesthesia, whereas TDC was primarily performed under local anesthesia with 1% lidocaine with epinephrine (1:100,000). The thickest portion of the subdural collection, typically the parietal boss, was chosen for the location of the craniostomy.

**FIGURE 1:**
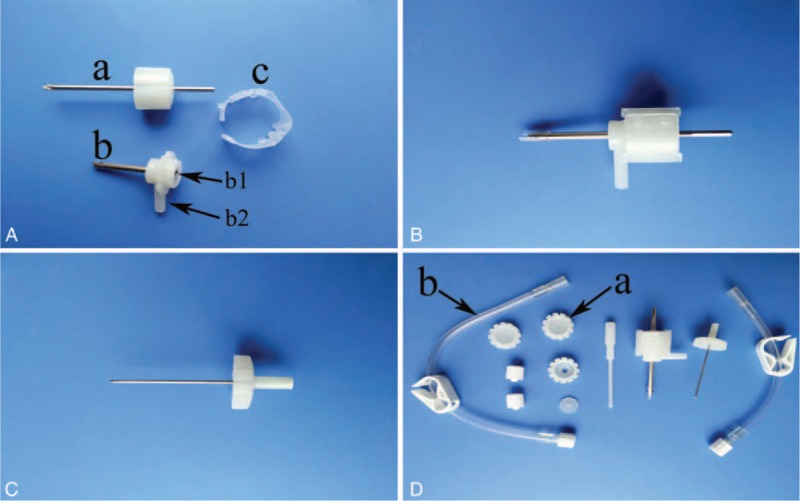
(A) Core needle (a), sleeve (b), and plastic sheet (c). Port b1 = irrigation using the spray needle. Port b2 = drainage. (B) YL-1 puncture needle. (C) Spray needle. (D) Seal cover for port b1 (a) and drainage tube (b).

The modified TDC technique, which was performed using a YL-1 puncture needle in our study, had been introduced by Zhang et al.^7^ The YL-I puncture needle was designed and is produced by Beijing WanTeFu Medical Apparatus Co., Ltd. (Beijing, China); the device is a trocar that consists of a sleeve and a core needle with a sawtooth tip. These 2 components are fixed together with a plastic sheet. A spray core needle, which features a closed tip and many small holes in the distal wall, is also used to eject water in all directions for irrigation (Figure 1). The YL-1 puncture needle is 3 mm in diameter and 2.5 cm in length. The needle can be shortened by adding silicone gaskets.

**FIGURE 2:**
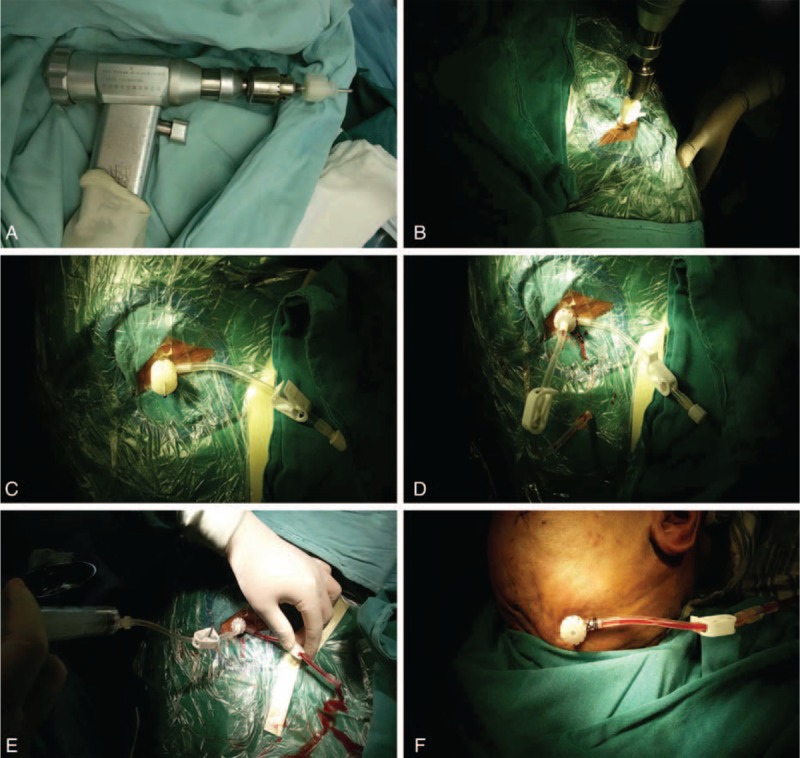
Modified TDC procedure using the YL-1 puncture needle. (A) Fixation of the YL-1 puncture needle in an electric drill handle. (B, C) Drilling into the hematoma cavity. (D) Removal of the core needle and insertion of the spray core needle. (E) Irrigation. (F) Closure of the irrigating port with the seal cover and drainage. TDC = twist drill craniostomy.

**FIGURE 3:**
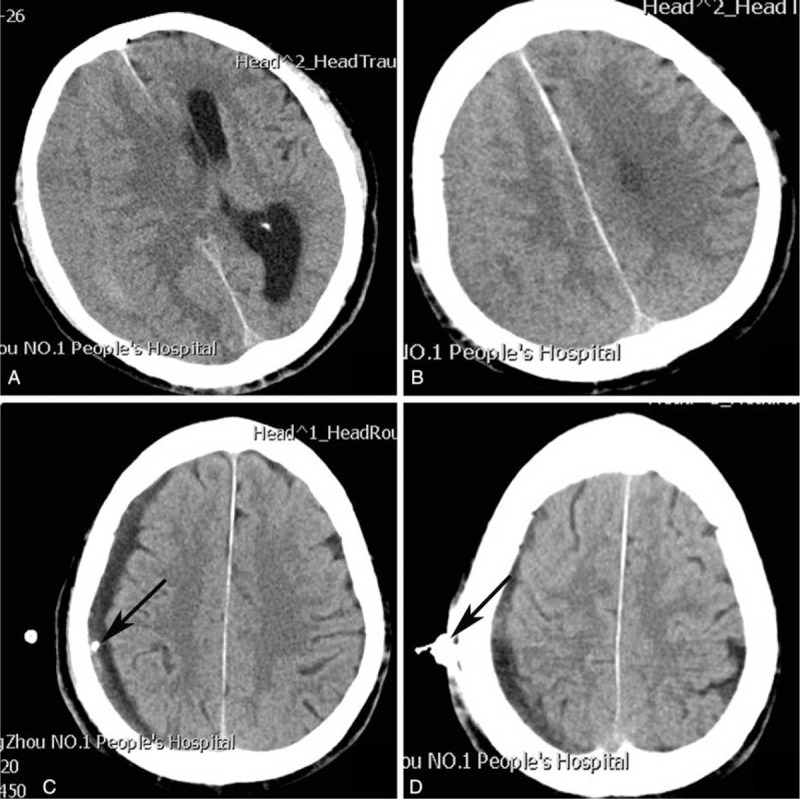
(A, B) Preoperative chronic subdural hematoma. (C, D) Postoperative modified TDC. Arrow = puncture needle, TDC = twist drill craniostomy.

The procedure was performed in steps. First, the needle was fixed in an electric drill handle, and the scalp was penetrated. The skull and dura were entered by drilling rapidly to reach the subdural cavity. The needle was automatically fixed by the skull. The core needle was removed to open 2 ports in the tail end: 1 for drainage and the other for irrigation. The spray core needle was inserted to irrigate the subdural hematoma cavity. A collection bag was connected to the drainage port, and the irritating port was closed (Figures 2 and 3).

For BHC, a 3- to 4-cm incision was made on the skin, a burr hole (1-cm diameter) was drilled, a catheter was inserted into the cavity, and the subdural hematoma was washed with sterile saline until the irrigation fluid ran clear. Finally, the catheter was indwelled for drainage and connected to a collection bag.

The drainage duration was based on the drain output and CT or MRI scan appearance. This drainage period ranged from 1 to 3 days.

### Outcome Measures

In the present study, the neurological status was evaluated according to the Markwalder grading system^8^ before surgery (at admission) and after surgery (at discharge or at the performance of a further procedure for complications), and grade 0 was set as a good outcome. The CSDH resolution was graded depending on rehabilitation of the midline and hematoma size, by comparing the CT scans before and after the procedure (Table 1).

**TABLE 1:**
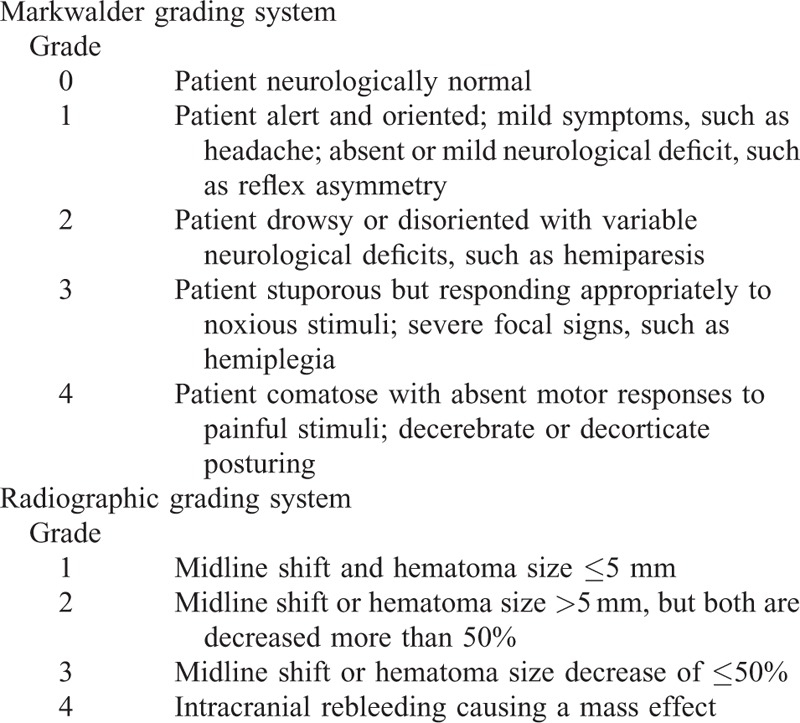
Outcome Evaluation System

### Follow-Up

All patients were instructed to complete follow-up via an outpatient review 2 to 4 weeks following discharge. Neurological and radiographic examinations were performed during the review. In the absence of abnormal events (such as headache, weakness, cognitive decline, or other neurologic disorders), the patients were no longer followed; patients with residual symptoms or an asymptomatic recurrence of CSDH were requested to attend a further follow-up visit after 2 to 4 weeks or at a time determined by the patient, according to their condition.

### Statistical Analysis

Based on whether the distribution was normal or non-normal, continuous variables were expressed as the mean ± standard deviation or median (interquartile range), and differences between variables were evaluated using an independent samples *t* test or rank-sum test. Categorical variables were expressed as a number (%), and differences between variables were compared using the chi-squared test or Fisher exact test. The level of significance was set at *P* < 0.05 for all tests. The statistical analyses were performed using SPSS 17.0 (SPSS, Chicago, IL).

## RESULTS

### Baseline Characteristics Between the BHC and TDC Groups

Most of the baseline characteristics, except anesthesia, did not significantly differ between the 2 groups (Table 2). General anesthesia was adopted in 42 (79.2%) patients in the BHC group and in 1 (1.5%) patient in the TDC group (*P* < 0.001). The preoperative neurological status was similar in patients from the BHC and TDC groups (*P* = 0.852). The preoperative CT scans revealed that most CSDHs were unilateral (84.9% in the BHC group and 82.4% in the TDC group, *P* = 0.708) and frontotemporoparietal (45.3% in the BHC group and 44.1% in the TDC group). The preoperative sizes of the hematoma and the midline shift were similar between the BHC and TDC groups. Typical symptoms in the patients with CSDHs included weakness, headache, and cognitive decline; 2 patients in the BHC group were stuporous but responded appropriately to noxious stimuli; no seizures or comas were observed in either group. Weakness was the most frequent symptom (100% in the BHC group and 98.5% in the TDC group), and cognitive decline was relatively infrequent (30.2% in the BHC group and 22.1% in the TDC group). Postoperative death or neurological infection was not observed in either group.

**TABLE 2:**
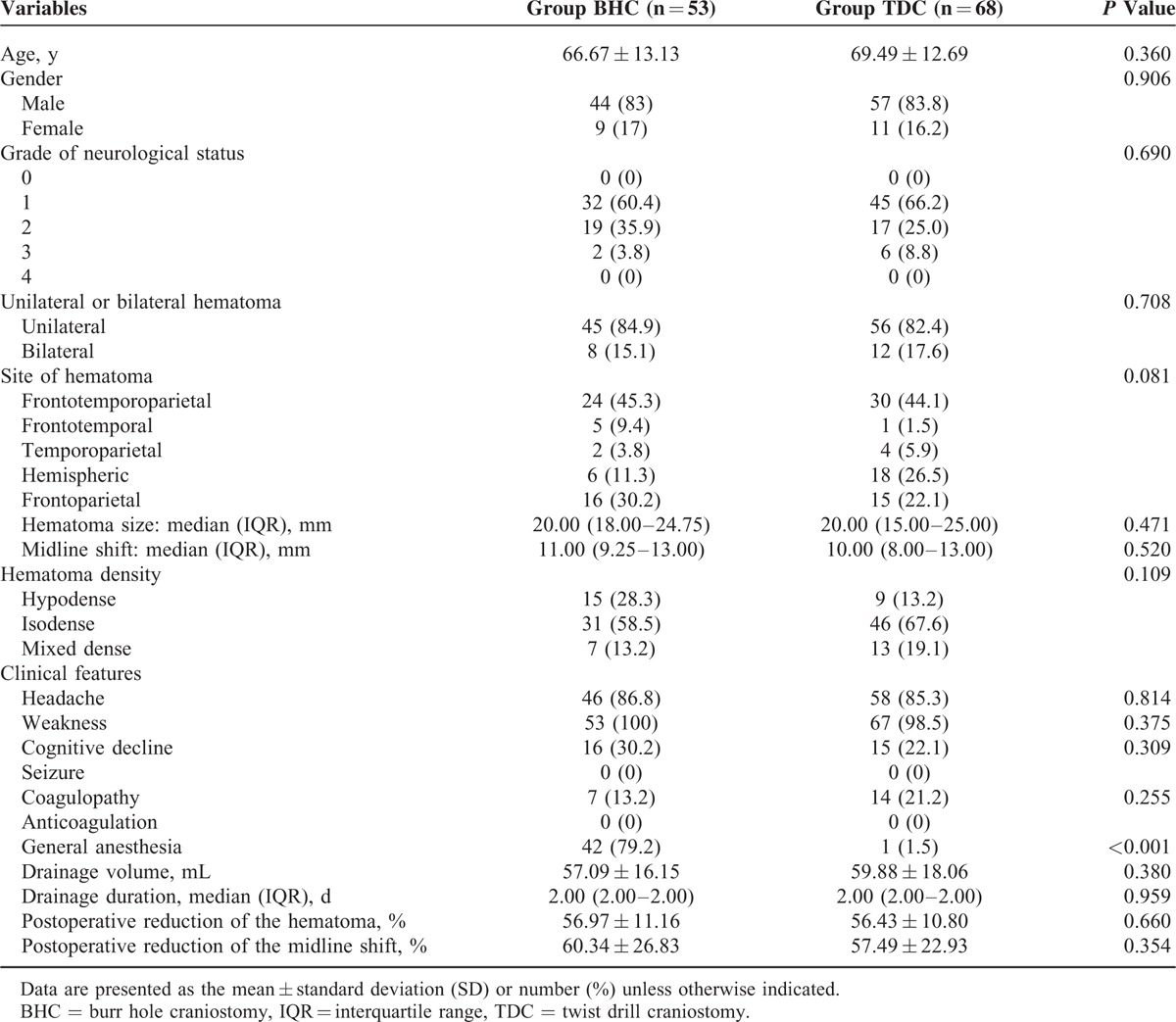
Baseline Characteristics of the Patients in Group BHC and Group TDC

### Neurological Outcome

The neurological status, as presented in Table 3, was significantly improved by surgery in the patients from the BHC (*P* < 0.001) and TDC (*P* < 0.001) groups. Although 3 patients in the BHC group had a grade of 3 with secondary acute intracranial hematoma, the postoperative neurological status did not differ significantly between the BHC and TDC groups (*P* = 0.852). The good outcome (grade 0) for patients in the BHC and TDC groups was 66.0% and 66.2%, respectively.

**TABLE 3:**
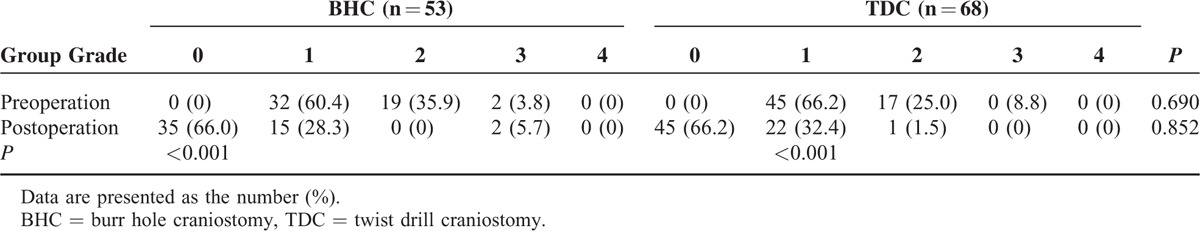
Primary Outcome

### Radiological Outcome

Radiological improvement indicated by a postoperative CT or MRI was found in all patients in both groups, with the exception of the 3 patients with secondary acute intracranial hemorrhage in the BHC group. Compared with preoperation, the postoperative radiological status improved significantly in patients from both the BHC and TDC groups (*P* < 0.001), while the difference between the 2 groups did not reach significance (*P* = 0.264; Table 4).

**TABLE 4:**
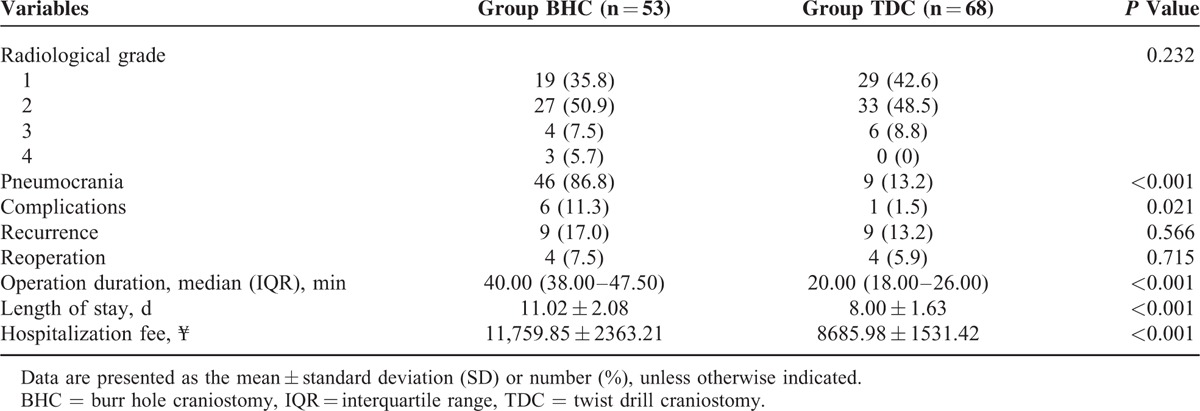
Secondary Outcomes

### Postoperative Complications

**FIGURE 4:**
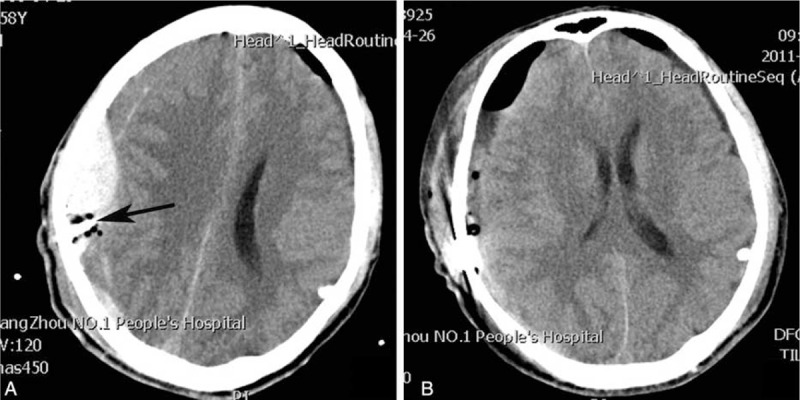
(A) Acute epidural hematoma secondary to BHC. (B) Postoperation of the craniostomy. Arrow = drainage catheter, BHC = burr hole craniostomy.

In the BHC group, 2 patients developed acute epidural hematomas and 1 acute subdural hematoma around the burr hole site; these patients underwent further surgical management (Figure 4). Three patients (male, ages 73, 81, and 84, respectively) in the BHC group developed pneumonia, which was cured using antibiotics. In the TDC group, the brain of 1 patient had been penetrated beneath the twist drill site and suffered mild contusion and laceration, which was cured using nonsurgical treatment. Seizures or neurological infections were not observed in either group. The complications in the BHC group were more severe and more frequent than those in the TDC group, and the difference was significant (*P* = 0.021; Table 4).

### Recurrence of CSDH

CSDH recurrence was observed in patients of both groups: 9 instances (13.2%) in the TDC group with 4 (5.9%) reoperative cases and 9 instances (17.0%) in the BHC group with 4 (7.5%) reoperative cases. The recurrence rate did not significantly differ between the 2 groups (recurrence, *P* = 0.566 and reoperation, *P* = 0.715; Table 4).

### Pneumocrania

Pneumocrania was found in 46 (86.8%) patients in the BHC group and 9 (13.2%) patients in the TDC group. The difference was significant (*P* < 0.001; Table 4).

### Operation Duration and Length of Stay

The modified TDC surgery required significantly less time to complete (20.00 min, with a range of 18.00–26.00 min) than the BHC procedure (40.00 min, range 38.00–47.50 min; *P* < 0.001). The length of hospital stay for TDC patients was 8.00 ± 1.63 days (range 4–12 days), which was significantly shorter than that for BHC patients (11.02 ± 2.08 days; range 7–16 days; *P* < 0.001; Table 4).

## DISCUSSION

### Technical Advantages of Our Modified TDC

TDC was first introduced by Tabaddor and Shulmon^9^ and has been repeatedly described in subsequent studies.^2,10^ Various modifications have made TDC less invasive, safer, and simpler to perform. Emonds and Hassler^11^ modified TDC using a new device (a hollow screw), which was fixed into a drilled hole in the skull and connected with a closed drainage system with a collection bag. The hollow screw used by Krieg et al^12^ had an inner diameter of 3 mm and an outer diameter of 5 mm (Teleflex Medical, Belp, Switzerland). Another similar system, the subdural evacuating port system, was used to modify TDC. This system utilizes a bulb suction apparatus with gentle negative pressure to promote brain re-expansion.^13^ Brain penetration was associated with TDC because it was a “blind” technique.^6^ To prevent brain penetration, Sucu et al^6^ and Yadav et al^14^ modified TDC by adjusting the drilling angle. By contrast, Reinges et al^15,16^ modified TDC with a self-controlling, preadjustable mechanical twist drill trephine. They drilled only the skull and perforated the dura and the outer neo-membrane of the hematoma with a 14G Teflon cannula, removed the cannula after the cessation of CSDH efflux, and closed the skin with a single suture and no irrigation. Mostofi and Marnet^17^ similarly modified TDC and used a needle (trocar) that was 1.1 mm in diameter and 30 mm in length. They pierced the dura with the needle, evacuated the hematoma with a 20-mL syringe in several steps, and did not irrigate the site.

The various TDC techniques mentioned above share 2 important and independent steps: drilling the skull and then opening or perforating the dura for drainage. These procedures are relatively complex and susceptible to pneumocrania, which increases the risk of recurrence.^18,19^ The present modified technique with the YL-1 puncture needle overcame this disadvantage because the drill bit consists of a drainage tube and a core needle. Thus, drilling and drainage are seamlessly united into 1 step that is simple to perform and insusceptible to pneumocrania; we found in the present study that, compared with BHC, the modified TDC took far less time and caused far less pneumocrania. In addition, the modified TDC poses 5 additional advantages: the self-controlling preadjustable drill bit can effectively prevent brain penetration; 2 ports in the tail end are convenient for irrigation and drainage; the drill bit, which features a pointed tip, can rapidly drill through the skull and dura to prevent the separation of the dura mater from the skull, which has been thought to cause epidural bleeding^6^; the puncture needle can be fixed tightly to the diploe and prevent diploic bleeding; and a percutaneous puncture replaces scalp incision to minimize invasion.

### Efficacy of TDC and BHC

The efficacy of TDC and BHC has been extensively studied by evaluating multiple factors, mainly including neurological and radiological outcomes, mortality, complications, and recurrence. In 2010, Lega et al^5^ systematically reviewed 1491 patients who underwent TDC and 6222 patients who underwent BHC. In the TDC group, 61.2% of the patients were cured, 3.1% died perioperatively, 4.4% experienced nonfatal complications, and 31.3% experienced recurrence; in the BHC group, 80.8% of the patients were cured, 2.5% died perioperatively, 6.2% experienced nonfatal complications, and 10.5% experienced recurrence.

Ducruet et al^1^ reported a systematic review of 45 studies in 2012. Of the patients who underwent TDC, 88% had good outcomes, 67% to 86% exhibited neurologic improvements, 0% to 18% experienced complications, 3% to 33% experienced recurrence, and 8% to 26% underwent reoperation; the mortality rate was 5% to 8%. Of the patients undergoing BHC, 58% to 90% had good outcomes, 69% to 88% exhibited neurologic improvements, 0% to 25% experienced complications, 2% to 31% experienced recurrence, and 8% to 33% underwent reoperation; the mortality rate was 0% to 13%. The results of a meta-analysis indicated good outcomes or neurologic improvement for 93.5% and 84.9%, complications for 2.5% and 9.5%, recurrence or reoperation for 28.1% and 11.7%, and mortality for 5.1% and 3.7% of TDC and BHC patients, respectively.

The outcomes of this study were excellent compared with those of previously published studies: in the patients who underwent modified TDC and BHC, the good-outcome (being cured) rates were 66.2% and 66.0%, respectively; the complication rates were 1.5% and 11.3%, respectively; the recurrence rates were 13.2% and 17.0%, respectively; the reoperation rates were 5.9% and 7.5%, respectively; and no perioperative deaths were observed.

### Modified TDC vs BHC

#### Surgery Efficacy

Regarding the neurological outcome in the present study, the surgery efficacy of the modified TDC was not significantly different from that of BHC (*P* = 0.852), indicating that the modified TDC was as effective as BHC. In addition, the radiological outcomes between the 2 groups were not significantly different (*P* = 0.232), indicating that the modified TDC was as effective as BHC for hematoma drainage. Horn et al^2^ compared 55 patients undergoing TDC with 24 patients undergoing BHC in a prospective nonrandomized trial and concluded that TDC is as effective as BHC for treating CSDH. Muzii et al^3^ conducted a prospective study comparing 22 patients undergoing TDC and 24 patients undergoing BHC and stated that TDC was at least as effective as BHC. Similar studies also reached the same conclusion.^9–10^ Interestingly, a more-optimistic conclusion was reported by Smely et al,^20^ who prospectively compared 33 patients undergoing TDC with 33 patients undergoing BHC and concluded that TDC was superior to BHC for treating CSDH.

#### Complications

In our study, the complication rate for the TDC group was significantly lower than that for the BHC group (*P* = 0.021), which indicated that the modified TDC was safer than BHC. In the TDC group, only 1 patient experienced complications; this patient's brain was penetrated, which may have occurred because the puncture site was too close to the edge of the collection site. By contrast, in the BHC group, 6 cases with complications were observed: 1 patient with acute subdural hematoma, 2 with acute epidural hematoma, and 3 with pneumonia. Epidural bleeding was caused by the separation of the dura mater from the skull due to a blunt drill bit tip.^21^ Subdural hematomas may occur due to bleeding of the dura mater and diploe, whereas the modified TDC of the present study was especially potent in preventing such complications due to previously described reasons. Elderly patients undergoing artificial ventilation during general anesthesia are susceptible to pneumonia; consequently, the modified TDC (performed mostly under local anesthesia) is superior to BHC (performed mostly under general anesthesia).

#### Pneumocrania and Recurrence

Pneumocrania, which was considered to be a risk factor of CSDH recurrence,^18^ was significantly rarer in patients undergoing the modified TDC compared with those undergoing BHC, indicating that the former was safer than the later, although CSDH recurrence did not differ significantly between the 2 groups because it was related to multiple factors. In addition to pneumocrania, Chon et al^21^ reported that postoperative midline shifting of ≥5 mm, diabetes mellitus, preoperative seizure, preoperative hematoma width of ≥20 mm, and anticoagulant therapy were independent predictors of recurrence. Tugcu et al^22^ found that bilateral hematoma was correlated with a high recurrence rate. Lin et al^23^ discovered that the mean hematoma density, the separated type, and bilateral and skull base involvement of CSDHs were significantly related to postoperative recurrence. Protective factors have also been explored in many studies, including artificial cerebrospinal fluid irrigation^19,24^ and postoperative drainage.^25,26^ Rychlicki et al stated that removing the hematoma fluid is important^27^; this fluid is rich in anticoagulative and fibrinolytic factors and is likely the cause of further hemorrhage. Sufficient hematoma evacuation and irrigation of the hematoma cavity are also important to prevent recurrence.^28^

#### Duration of Operation and Length of Hospital Stay

The modified TDC, primarily performed under local anesthesia and requiring an average of 20 min to complete (the drilling duration did not exceed 10 s, and irrigation occupied the remaining time), was simpler to perform compared with BHC, which was primarily performed under general anesthesia and required an average of 40 min. Elderly patients, especially those with co-morbidities, can benefit from simpler procedures because they reduce the perioperative risk. The average length of hospital stay in the TDC group was significantly shorter than that in the BHC group because TDC minimized the invasiveness of both surgery and anesthesia.

### Study Limitations

This study had certain limitations. First, it was a nonrandomized, retrospective study. Therefore, the authors cannot deny the possibility of a selection bias for the surgical technique. Second, most patients were followed up for fewer than 4 weeks; thus, the surgical efficacy could not be accurately evaluated. This study examined a relatively small patient cohort with a relatively weak potential to represent the CSDH population. Hence, further randomized research based on a large population and appropriate follow-up durations are required to provide more information about CSDH.

## CONCLUSION

Modified TDC with a YL-1 puncture needle is a minimally invasive surgical technique to treat CSDH that is as effective as BHC, but safer and simpler than BHC; this modified TDC should be considered for patients with CSDH, especially the elderly.
